# Molecular Basis of Intron Retention in *PI-PLC γ1* mRNA from Osteoarthritis Synoviocytes

**DOI:** 10.3390/ijms26178123

**Published:** 2025-08-22

**Authors:** Alessia Mariano, Daniel D’Andrea, Roberto Mattioli, Sergio Ammendola, Anna Scotto d’Abusco

**Affiliations:** 1 Department of Biochemical Sciences, Sapienza University of Rome, P.le Aldo Moro, 5, 00185 Rome, Italy; alessia.mariano@uniroma1.it (A.M.); roberto.mattioli@uniroma1.it (R.M.); 2 School of Engineering Mathematics and Technology, University of Bristol, Bristol BS8 1TW, UK; daniel.dandrea@bristol.ac.uk; 3 Ambiotec di Sergio Ammendola, Via Appia Nord 47, 04012 Cisterna di Latina, LT, Italy; ammendola@ambiotec.it

**Keywords:** intron retention (IR), phosphatidyl inositol phospholipase C γ1 (PI-PLC γ1), osteoarthritis (OA), RNA-seq analysis, interindividual variability, CUG binding protein, Elav-Like Family 1 (CELF1)

## Abstract

Intron retention (IR) is one of the cellular mechanisms to perform alternative splicing and thus control gene expression in several mammalian cellular pathways. IR in *PI-PLC γ1* mRNA was observed in some primary synoviocyte samples from osteoarthritis (OA) patients, likely due to inter-patient variability. The aim of the present manuscript was to explore the *PI-PLC γ1* IR molecular mechanism as a consequence of nutraceutical treatment of synoviocytes and the molecular basis of individual response. To evaluate the gene expression modulation of molecules involved in mRNA splicing, an RNA-seq analysis was performed, and the transcription modulation of six differentially expressed genes was validated by RT-PCR. Moreover, through a silencing experiment, the relationship between *PI-PLC γ1* IR and the six modulated genes was explored. Finally, two of them, the RNA-binding proteins CELF1 and PTBP3, whose mRNA levels were elevated in samples exhibiting IR, were analyzed in detail. CELF1 and PTBP3 were overexpressed in synoviocytes lacking *PI-PLC γ1* IR, and we found that CELF1 was responsible for IR, whereas PTBP3 did not seem to be involved. In conclusion, in our experimental model, the role of CELF1 protein in *PI-PLC γ1* IR was explored, opening new scenarios for understanding the molecular mechanisms underlying the IR phenomenon present in several kinds of diseases.

## 1. Introduction

Intron retention (IR) is the phenomenon by which, during mRNA maturation, some introns remain unspliced in polyadenylated transcripts. IR is one of the mechanisms of alternative splicing that, initially, was mainly studied in plants, fungi, insects, and viruses [1]. More recently, the importance of IR in the regulation of gene expression in several biological processes has also been acknowledged in mammalian cells [2,3]. To date, IR has been considered a key mechanism able to control gene expression during development, differentiation, and activation of several mammalian cells, particularly those present in neuronal and hematopoietic systems [3,4,5]. Unlike other alternative splicing mechanisms, which generate new protein isoforms, IR predominantly results in post-transcriptional gene repression, due to the presence of stop codons in the retained introns. The premature termination codons facilitate the nonsense-mediated decay [6], or, if the unspliced mRNAs remain in the nucleus, the accumulated mRNA can be degraded by the RNA exosome [7]. Both cases result in decreased gene expression. In some cases, such as in *Drosophila melanogaster*, intron-retaining transcripts accumulated in the nucleus can be correctly spliced, obtaining a rapid protein synthesis in response to some kinds of stimulus, which leads to an enhancement of long-term memory [8]. Overall, IR can have diverse impacts on transcripts and proteins, and in turn on cell life, making it interesting to understand the role of this phenomenon in specific biological contexts. Previously, we described IR in the *PI-PLC γ1* mRNA, which leads to a decrease in this protein in fibroblast-like synovial (FLS) cells in an in vitro model of osteoarthritis (OA). Moreover, we found that IR made cells more responsive to treatment with *Harpagophytum procumbens* root extract (HPE) [9]. In particular, in FLSs showing IR in *PI-PLC γ1* mRNA, treatment with HPE led to the downregulation of metalloproteases (MMPs), MMP-3, MMP-13, and A disintegrin and metalloproteinase with thrombospondin motifs-5 (ADAMTS-5), involved in cartilage degradation in OA [10]. OA is the most common joint rheumatic disease, characterized by low-grade inflammation, degradation of articular cartilage, and, in turn, joint pain [11,12]. The degradation of articular cartilage is due to an imbalance between extracellular matrix (ECM) component deposition and secretion of MMPs involved in matrix turnover. To date, there has been no pharmaceutical therapy, and the disease is mainly treated with non-steroidal anti-inflammatory drugs and analgesic agents with the aim of alleviating the symptoms [13]. Nutraceuticals are also administered to treat OA, among which *Harpagophytum procumbens* extract has been used for a long time to counteract the pain associated with OA. Our previous findings demonstrated that HPE can downregulate MMP production, although its effectiveness was evident only in a subset of primary synoviocyte samples. In particular, HPE was effective in samples showing IR in *PI-PLC γ1*, thus suggesting that, in this case, the response to HPE treatment was due to inter-individual diversity regarding the splicing of *PI-PLC γ1* mRNA. Considering that PI-PLC γ1 decrease has been associated with ECM component synthesis and degradation in OA chondrocytes, the regulation of its expression through alternative splicing in synoviocytes and the mechanism of intron retention represent highly relevant and promising areas of study.

In the present manuscript, we performed RNA-Seq analysis on synoviocytes without IR (WT), with IR (IR), and with IR treated with HPE (IR_HPE_), aiming to explore the molecular mechanisms underlying IR, identify intracellular targets of HPE treatment, and clarify its effects on the key molecules involved in OA pathogenesis.

## 2. Results

### 2.1. PI-PLC γ1 Intron Retention: Background

The synoviocytes isolated from synovial membranes of several patients were analyzed for the presence of the IR phenomenon, using an oligonucleotide pair mapping within an exon and a nearby intron, as described in the Materials and Methods section (Section 4.2 paragraph). Almost 40% of the analyzed samples, obtained from 30 OA patients who underwent total knee or hip arthroplasty surgery, showed the retention of introns (Table S1, in Supplementary Materials). IR in *PI-PLC γ1* mRNA causes a decrease in PI-PLC γ1 protein, due to the presence of a stop codon inside the retained introns. Thus, the incorrectly spliced mRNA produces a very short nonfunctional protein [9].

In our previous studies, the cells were treated with HPE, an extract used in traditional medicine to treat OA, in order to analyze its effects on inflammatory processes. We observed that HPE treatment increased the retention of introns in those samples where the phenomenon was present. Moreover, a decrease in PI-PLC γ1, mainly after HPE treatment, induced a decrease in several markers of OA, such as MMPs [9]. Thus, further studies are needed to understand the mechanism of intron retention.

### 2.2. RNA-Seq Analysis

**Figure 1:**
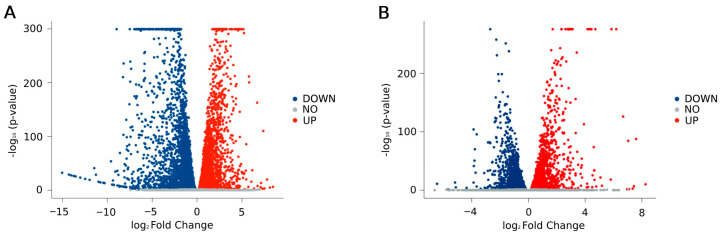
Volcano plots of differentially expressed genes: (**A**) WT (*PI-PLC γ1* mRNA correctly spliced) vs. IR (*PI-PLC γ1* mRNA with intron retention); (**B**) IR_HPE_ (*PI-PLC γ1* mRNA with intron retention treated with HPE) vs. IR. Reported are the negative log_10_-transformed adjusted *p*-values plotted against the log_2_ fold changes. The red dots represent the upregulated genes, the blue dots represent the downregulated genes, and the gray dots represent genes that were not statistically significant. Genes with adjusted *p*-values after Bonferroni correction < 0.05 were considered significantly modulated.

RNA-seq analysis was performed with the aim of identifying the factors potentially involved in IR. mRNA from a sample without intron retention, thus showing wild-type mechanism regarding the splicing of *PI-PLC γ1* mRNA (WT), a sample exhibiting intron retention in *PI-PLC γ1* mRNA (IR), and the same IR sample following HPE treatment (IR_HPE_) were analyzed. A comparison between the transcriptome data in WT and IR samples and IR_HPE_ and IR samples was performed, finding several differentially expressed mRNAs. Principal component analysis (PCA) showed a clear separation among the groups (Figure S1). To confirm our previously published results [9], we first examined the expression levels of the *PI-PLC γ1* gene in IR vs. WT and IR_HPE_ vs. IR samples (Figure S2). The analysis revealed that *PI-PLC γ1* was significantly downregulated in IR_HPE_ compared to IR (*p* = 0.00074), while no significant difference was observed between IR and WT (*p* = 0.24) (Figure S2), thus supporting our previous findings. The comparison between IR vs. WT samples showed 3608 upregulated genes and 3773 downregulated ones (Figure 1A). Regarding the comparison between IR_HPE_ vs. IR samples, 2213 genes were upregulated, and 1701 were downregulated (Figure 1B).

**Figure 2:**
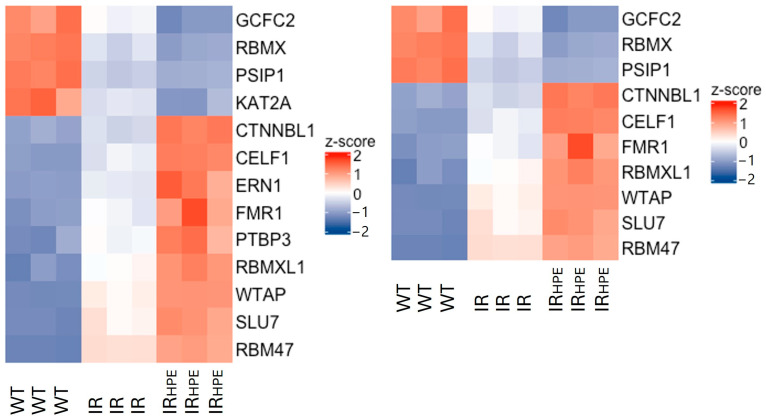
Heatmap showing the genes that were either upregulated in WT (*PI-PLC γ1* mRNA correctly spliced) vs. IR (*PI-PLC γ1* mRNA with intron retention) samples and downregulated in IR_HPE_ (*PI-PLC γ1* mRNA with intron retention treated with HPE) vs. IR samples (WT > IR, IR > IR_HPE_, decrease–decrease) or downregulated in WT vs. IR samples and upregulated in IR_HPE_ (WT < IR, IR < IR_HPE_, increase–increase) vs. IR samples and involved in RNA splicing (left, *n* = 13) or RNA splicing via transesterification reaction (right, *n* = 10). The Z scores of gene expression are depicted as a gradient from blue (low expression) to red (high expression) (*n* = 3 biological replicates).

Considering that our hypothesis was that the *PI-PLC γ1* intron retention was due to some alterations (either as up- or downregulation) in the splicing process in IR compared to WT, and that HPE treatment (IR_HPE_) could further affect the alterations, we focused on genes coding for proteins involved in splicing mechanisms. We selected genes that were either upregulated in WT vs. IR samples and downregulated in IR_HPE_ vs. IR samples (WT > IR, IR > IR_HPE_, decrease–decrease) or downregulated in WT vs. IR samples and upregulated in IR_HPE_ vs. IR samples (WT < IR, IR < IR_HPE_, increase–increase). Therefore, a list of 13 genes involved in RNA splicing (GO:0008380) and 10 genes involved in RNA splicing via transesterification reactions (GO:0000375) was generated, and a heatmap representation was created (Figure 2) (“The Gene Ontology knowledgebase in 2023” by The Gene Ontology Consortium (https://geneontology.org/, accessed on 11 April 2023)). Among those genes, after literature analysis, six characterized genes, involved in mRNA splicing or RNA splicing via transesterification reaction, were selected for further analyses.

### 2.3. RNA-Seq Validation Through RT-PCR

The selected six genes involved in splicing processes (*CELF1*, *WTAP*, *PSIP1*, *ERN1*, *RMB47*, and *PTBP3*) were analyzed at the mRNA level using RT-PCR to verify the consistency between the RNA-seq results and the mRNA levels of the modulated genes.

CUG binding protein, Elav-Like Family 1 (CELF1), is an RNA-binding protein that is associated with various mRNA metabolism processes, including pre-mRNA splicing, mRNA decay, and translation [14]. Wilms’ tumor 1-associating protein (WTAP) is a component of the m6A methyltransferase complex, and it is a regulatory subunit whose function is to recruit this complex to the target mRNA [15]. PC4 and SF2 interacting protein 1 (PSIP1) is a multifunctional chromatin protein that modulates alternative splicing [16]. RNA-binding motif protein 47 (RBM47) is a single-stranded RNA-binding protein that works in several RNA processes, such as alternative splicing, RNA stabilization, and RNA editing [17]. Endoplasmic Reticulum to Nucleus signaling 1 (ERN1) encodes for Inositol-Requiring Enzyme 1 alpha (IRE1α) protein kinase. The latter mediates the splicing and activation of the stress response transcription factor X-box binding protein 1 (XBP-1) [18]. Polypyrimidine tract binding protein 3 (PTBP3) is involved in post-transcriptional regulation as an RNA-binding protein. It mediates pre-mRNA alternative splicing regulation and acts upstream of or within the negative regulation of mRNA splicing, via the spliceosome [19].

**Figure 3:**
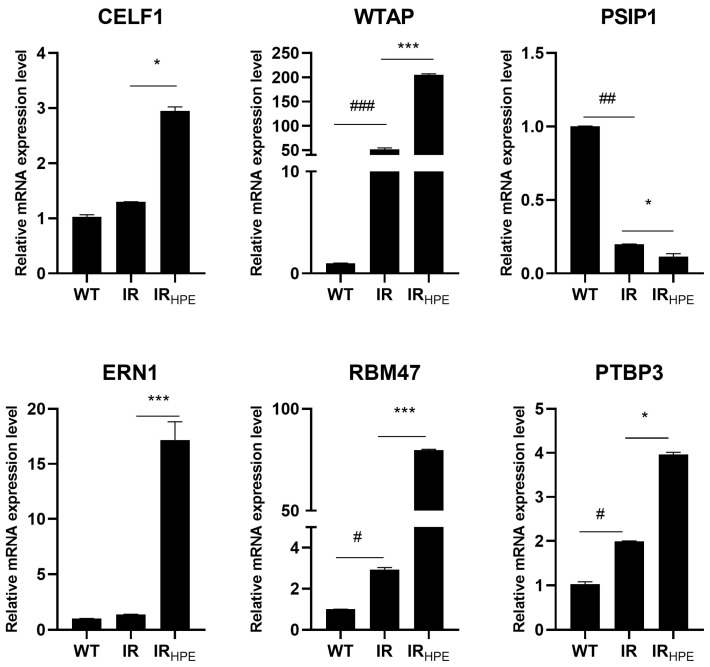
Validation of RNA-seq results through RT-PCR. mRNA was extracted from untreated cells with intron retention (IR) or treated with 0.1 mg/mL HPE_DMSO_ for 24 h (HPE_IR_) and untreated cells without intron retention (WT), and then analyzed by RT-PCR. Gene mRNA amount was normalized with respect to 18S (2^−ΔΔCt^ method). CUG binding protein, Elav-Like Family 1 (CELF1), Wilms’ tumor 1-associating protein (WTAP), PC4, and SF2 interacting protein 1 (PSIP1), Endoplasmic Reticulum to Nucleus signaling 1 (ERN1), RNA binding motif protein 47 (RBM47), and polypyrimidine tract binding protein 3 (PTBP3). Results are expressed as mean ± SD of data obtained by three independent experiments. * *p* < 0.05 HPE_IR_ vs. IR; *** *p* < 0.005 HPE_IR_ vs. IR; # *p* < 0.05 IR vs. WT; ## *p* < 0.01 IR vs. WT; ### *p* < 0.005 IR vs. WT.

The results of RT-PCR analysis for these six genes were in agreement with the RNA-seq results, indicating that the RNA-sequencing results were consistent (Figure 3).

Moreover, the modulation of these genes was confirmed in other samples showing intron retention (IR), IR treated with HPE (IR_HPE_), and samples showing correct splicing (WT), which had not been analyzed by RNA-seq.

**Figure 4:**
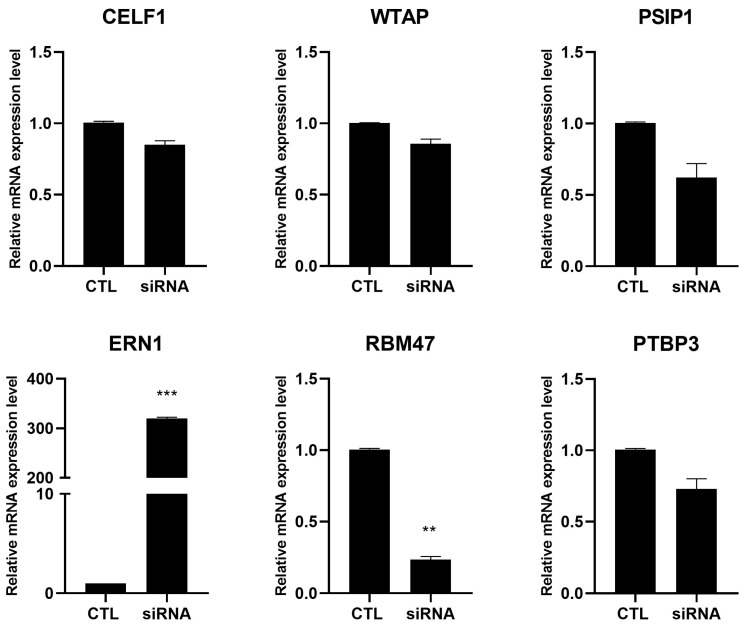
Effects of *PI-PLC γ1* silencing on *CELF1*, *WTAP*, *PSIP1*, *ERN1*, *RBM47*, and *PTBP3* gene expression compared with non-silenced cells (CTLs). Cells were analyzed after 24 h of silencing. The mRNA was extracted and analyzed by RT-PCR. mRNA amount was normalized with respect to 18S (2^−ΔΔCt^ method). Results are expressed as mean ± SD of data obtained by three independent experiments. ** *p* < 0.01 silenced vs. CTL; *** *p* < 0.005 silenced vs. CTL.

To understand whether the modulation of these genes affected the *PI-PLC γ1* mRNA splicing or whether the *PI-PLC γ1* intron retention, and thus the decrease in PI-PLC γ1 protein, affected their expression, a silencing experiment was performed in cells showing correct splicing. The findings showed that following *PI-PLC γ1* silencing via siRNA, *WTAP*, *CELF1*, *PSIP1*, and *PTBP3* were not modulated, suggesting that their modulation could be responsible for *PI-PLC γ1* intron retention or completely independent of this phenomenon. Regarding *ERN1*, it was increased, and *RBM47* was decreased after *PI-PLC γ1* silencing; thus, their modulation is dependent on PI-PLC γ1 depletion (Figure 4).

**Figure 5:**
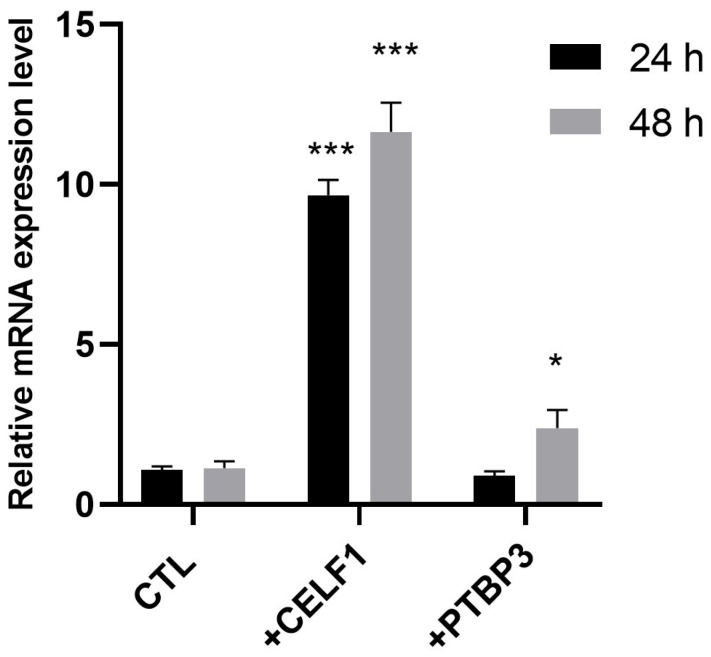
Effect of CELF1- and PTBP3-expressing vector transfections on *PI-PLC γ1* intron retention. Cells were left untreated (CTL) or transfected with CELF1-expressing vector (+CELF1) or with PTBP3-expressing vector (+PTBP3) for 24 h and 48 h. After transfection, mRNA was extracted, and *PI-PLC γ1* intron retention was analyzed by RT-PCR using two oligonucleotides, one mapping into an exon and the second one into an intron, as described in Figure 1. mRNA amount was normalized with respect to 18S (2^−ΔΔCt^ method). Results are expressed as mean ± SD of data obtained by three independent experiments. * *p* < 0.05 PTBP3 transfected vs. CTL; *** *p* < 0.005 CELF1 transfected vs. CTL.

Considering that the *PI-PLC γ1* silencing did not affect the modulation of *CELF1*, *WTAP*, *PSIP1*, and *PTBP3*, their modulation observed in cells presenting IR and in cells presenting IR and treated with HPE should be considered a characteristic of these cells and could be responsible for the observed IR. To verify whether their modulation was responsible for IR, we transfected cells not showing IR with expression vectors containing the gene coding for CELF1 or for PTBP3. The choice fell on these two proteins due to their role; they are both RNA-binding proteins. CELF1 is involved in both pre-mRNA splicing and translational processes by binding mRNA, and PTBP3 is involved in post-transcriptional regulation as an RNA-binding protein [20,21]. After transfection with CELF1- and PTBP3-expressing vectors, intron retention was analyzed by performing RT-PCR, as described above. The findings showed that the increased expression of CELF1 induced an increase in intron retention in *PI-PLC γ1* mRNA after both 24 h and 48 h transfection (Figure 5). Regarding the transfection with the PTBP3 expression vector, a slight increase in *PI-PLC γ1* intron retention was observed only at 48 h post-transfection, although to a lesser extent compared to CELF1 (Figure 5).

## 3. Discussion

Intron retention is one of the mechanisms able to promote the alternative splicing of polyadenylated mRNA. In turn, the alternative splicing is responsible for the protein variability inside the cells. Recently, intron retention has been described to be involved in some inflammatory disorders, such as idiopathic inflammatory myopathy, lupus erythematosus, and Alzheimer’s disease [22,23,24]. Previously, we described the IR phenomenon in the *PI-PLC γ1* mRNA in OA synoviocytes, resulting in a decrease in PI-PLC γ1 protein production, due to the presence of a stop codon within the first retained intron [9]. The IR in *PI-PLC γ1* concerns an mRNA region containing five introns, which are all retained when the phenomenon is present (Ensembl Gene: PLCG1 ENSG00000124181, 2023). Our previous interesting findings showed that, in those samples where the IR phenomenon was present, cells were more responsive to *Harpagophytum procumbens* root extract (HPE) [9]. OA is a joint degenerative disease due to an imbalance between the deposition of extracellular matrix (ECM) components, such as collagen type II, and the secretion of enzymes, such as metalloproteases, that degrade ECM components [25]. Currently, OA is an incurable disease, which is treated with symptomatic drugs, as steroidal or non-steroidal drugs, or with chondroprotective molecules, such as glucosamine and chondroitin sulfate. Herbal extracts are also administered to OA patients, among which the HPE has long been used in traditional medicine for its analgesic and anti-inflammatory properties [26,27]. Previously, we found that OA synoviocytes treated with HPE showed an increase in endocannabinoid receptor type 2 (CB2), thus explaining the analgesic effects of this extract [28]. Moreover, we found that HPE was able to induce a decrease in MMP-3, -13, and ADAMTS-5, which are the major factors responsible for the cartilage degradation in OA, but only in some human synoviocyte samples [9]. Furthermore, we found that the HPE-inhibited metalloprotease production was due to the *PI-PLC γ1* intron retention phenomenon, present only in some hFLSs, and that the HPE treatment was able to increase this kind of alternative splicing. In particular, we found the *PI-PLC γ1* IR in almost 40% of the analyzed synoviocyte samples, thus suggesting the presence of interindividual variability, leading to different responses to treatment administration. To date, the mechanism underlying IR in *PI-PLC γ1* mRNA has not been elucidated. The aim of the present study was to highlight the molecular mechanisms responsible for *PI-PLC γ1* IR. The strategy was to analyze, using RNA-seq, synoviocyte extracts from a sample showing correct splicing (WT), a sample showing *PI-PLC γ1* IR (IR), and a sample showing IR treated with HPE (IR_HPE_). Comparative analysis of the three samples revealed extensive modulation of mRNA expression profiles. Consequently, we focused our investigation on transcripts encoding proteins involved in mRNA splicing. In particular, we focused on those mRNAs that were increased in the IR sample compared to WT and further increased in the IR_HPE_ sample, or on those that were decreased in the IR sample compared to WT and further decreased in the IR_HPE_ sample. The chosen mRNAs encoded for CUG-binding protein, Elav-Like Family 1 (CELF1), Wilms’ tumor 1-associating protein (WTAP), PC4 and SF2 interacting protein 1 (PSIP1), Endoplasmic Reticulum to Nucleus signaling 1 (ERN1), RNA binding motif protein 47 (RMB47), and polypyrimidine tract binding protein 3 (PTBP3) [14,15,16,17,18,19]. The modulation of these mRNAs was confirmed by RT-PCR, and we found that their modulation was in line with RNA-seq results. *CELF1* and *ERN1* were slightly modulated in the IR sample compared to the WT sample, whereas they were statistically modulated after HPE treatment compared to both WT and IR samples. The other mRNAs were statistically modulated in the IR and IR_HPE_ samples compared to the WT sample. To verify whether the modulation of these mRNAs was due to *PI-PLC γ1* mRNA IR or whether they were responsible for the intron retention of *PI-PLC γ1* mRNA, their modulation was measured in the WT sample after silencing *PI-PLC γ1*, finding that *ERN1* and *RMB47* were modulated, whereas *CELF1*, *WTAP*, *PSIP1*, and *PTBP3* were not modulated. This result indicated that *ERN1* and *RMB47* were modulated as a consequence of PI-PLC γ1 decrease due to IR phenomenon. This is a noteworthy finding, mainly because ERN1 is involved in the modulation of collagen type II, a marker of both healthy and OA cartilage. Our unpublished preliminary results showed that the upregulation of ERN1 induced an increase in collagen type II production in human primary chondrocytes. With regard to CELF1, WTAP, PSIP1, and PTBP3, it can be concluded that they were either directly involved in regulating intron retention in *PI-PLC γ1* mRNA or that their expression was modulated independently of *PI-PLC γ1* IR. Considering that RNA-binding proteins CELF1 and PTBP3 are directly involved in mRNA binding and splicing, we decided to focus our attention on them. Synoviocytes showing correct splicing were transfected with an expression vector coding for CELF1 or for PTBP3 to investigate the overexpression of these proteins, and the findings showed that CELF1 could affect the splicing of *PI-PLC γ1* mRNA, considering that its overexpression induced *PI-PLC γ1* mRNA intron retention in the WT sample, whereas the overexpression of PTBP3 showed only a slight degree of *PI-PLC γ1* mRNA intron retention; thus, it could be not involved. In recent years, a growing body of evidence suggested that RNA-binding proteins (RBPs) play crucial roles in OA [19]. The RBPs involved in OA have shown functions in several intracellular mechanisms, such as in regulating gene transcription, RNA splicing, RNA stability and translation. Among others, FUS was reported to regulate gene transcription by working with RUNX2 to mediate transcription of collagen type X, which is a marker of hypertrophic differentiation in chondrocytes [29]. Moreover, FUS was described to be involved in splicing of *PDE4B* pre-mRNA to produce the circPDE4B, preventing the articular cartilage degeneration [30]. mRNA stability is prevalently due to N6-methyladenosine modification, involving several methyltransferases such as METTL3/14 and WTAP [19]. METTL3/14 has been described to mediate the methylation of transcription factor *SOX9* mRNA, disrupting its stability and thereby inhibiting the expression of collagen type II [31]. To the best of our knowledge, this is the first study suggesting a potential involvement of CELF1 in *PI-PLC γ1* splicing process. CELF1 is an RNA-binding protein belonging to the CELF family, which includes six genes in mammals [32]. These proteins present RNA recognition motifs (RRMs), and their role is to bind pre-mRNA affecting splicing [32]. Even if a tight consensus sequence for CELF protein binding sites is lacking, it has been described that CELF1 binds with high affinity mRNA regions rich in UGU trinucleotide [33] and often localized at exon–intron boundaries [14]. Our hypothesis is that CELF1 can bind *PI-PLC γ1* mRNA inhibiting the correct splicing, thus producing an improperly spliced mRNA retaining introns, which in turn, due to the presence of stop codons, produce truncated not working PI-PLC γ1 protein [9]. Our preliminary and unpublished findings showed that the samples presenting *PI-PLC γ1* intron retention had a moderate increase in *CELF1* mRNA level, whereas the samples showing *PI-PLC γ1* correct splicing did not show *CELF1* increase. Interestingly, a decrease in PI-PLC γ1 has been shown to be responsible for metalloprotease decrease, mainly after HPE treatment in OA synoviocytes [9].

In conclusion, we can hypothesize that the IR phenomenon regarding *PI-PLC γ1* mRNA could be due, at least in part, to the upregulation of CELF1. More studies are required to explore this hypothesis in more detail, in particular by conducting mRNA-binding protein experiments and investigating the phosphorylation status of CELF1, which could be induced by HPE treatment. On the other hand, we plan to gain more insight into the modulation of CELF1, both at mRNA and protein levels, in joint tissues isolated by a larger number of OA patients, in order to further confirm its involvement in the intron retention mechanism. Finally, considering that HPE inhibited metalloprotease production only in those OA synoviocytes presenting *PI-PLC γ1* mRNA intron retention, it could be useful to further study this aspect to verify its involvement in OA progression.

## 4. Materials and Methods

### 4.1. Synoviocyte Isolation and Cell Culture

Human primary fibroblast-like synoviocytes (FLSs) were isolated from synovial membranes obtained from 30 OA patients who underwent total knee or hip arthroplasty surgery. The study was approved by the Research Ethics Committee, ASL Lazio 2 (#005605/2019, 3 March 2019), and full ethical consent was obtained from all donors. The synovial membrane fragments, after mincing, were treated with 1 mg/mL collagenase type IV and 0.25% trypsin for 2 h, at 37 °C in agitation. Isolated cells were grown to 80% confluence in Dulbecco’s Modified Eagle Medium (DMEM) (HyClone, Logan, UT, USA) supplemented with L-glutamine, penicillin/streptomycin (Sigma-Aldrich, Co., Saint Louis, MO, USA) and 10% fetal bovine serum (FBS) and cultured at 37 °C and 5% CO_2_. The cells were characterized as synoviocytes type B for the presence of vimentin [34]. All experiments were carried out with synoviocytes at first passage (p1), isolated from at least 3 different donors.

### 4.2. Characterization for PI-PLC γ1 Intron Retention

**Figure 6:**
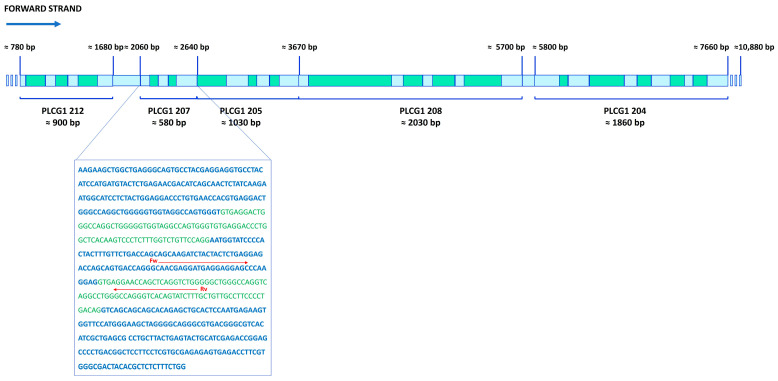
Schematic representation of the *PI-PLC γ1* gene with retained introns. *PI-PLC γ1*-encoding gene (10,880 bp) is shown with the five regions containing the retained introns in green. The sequence reported in the frame corresponds to the PLCG1-207 region, described by the HAVANA group at the Wellcome Trust Sanger Institute (Cambridge, UK) and sequenced in our lab. Exon sequences are shown in blue, and intron sequences are shown in green. The red arrows, Fw and Rv, represent the position and sequences of RT-PCR primers.

Previously, in our lab, the intron retention (IR) phenomenon was described in the phosphoinositide-specific phospholipase C γ1 (PI-PLC γ1) mRNA through semi-quantitative PCR [9]. In the present study, in order to characterize the synoviocytes for the presence of IR phenomenon, we designed two oligonucleotides: the forward one, PLC γ1fw (207), mapping in an exon (DQ297143.1, 5′-AACGAGGATGAGGAGGAG-3′) and the reverse one, PLC γ1rv (207), in an intron (DQ297143.1, 5′-AAAGATACTGTGACCCTGGC-3′) (Figure 6). RT-PCR amplification reveals the presence of intron-retaining transcripts with detectable product emerging around threshold cycle 30. In contrast, correctly spliced mRNA species are typically detected at later cycles, around threshold cycle 37.

### 4.3. Cell Treatment

Cells were treated for required times with 0.1 mg/mL of *Harpagophytum procumbens* root extract (HPE) dissolved in DMSO (HPE_DMSO_), and untreated cells were used as a control (CTL). Standardized HPE powder, containing 1.2% harpagoside, was provided by ACEF S.p.a. (Piacenza, Italy), and HPE_DMSO_ was previously characterized [34]. Solvent alone was also tested. Experiments were independently repeated at least three times.

### 4.4. RNA Extraction

Total RNA from untreated and treated FLSs was digested with DNaseI and then extracted with a blood/tissue total RNA extraction kit (Fisher Molecular Biology, Trevose, PA, USA). Reverse transcription was performed according to the manufacturer’s instructions with Meridian Bioscience Reverse Transcriptase (Bioline reagent Ltd., London, UK), using 150–180 ng/μL RNA. The quality control of RNA was assessed using agarose gel and spectrophotometer measures, verifying the 260/280 ratio.

### 4.5. RNA-Seq Library Preparation and Sequencing

The RNA concentration was determined using the Qubit RNA HS Assay Kit on the Qubit 3.0 Fluorometer (Thermo Fisher Scientific, Waltham, MA, USA). Each sample was used at a concentration of 50 ng/μL. Quality control was assessed by Labchip GX Touch HT instrument on DNA 5K/RNA/CZE Chip (Perkin Elmer, Thermo Fisher Scientific, Waltham, MA, USA) with RNA Pico Sensitivity Assay Reagents (Perkin Elmer). NextFlex PolyA beads 2.0 Kit and NextFlex Rapid Directional RNA-seq Kit 2.0 with UDIs (Perkin Elmer) were used for mRNA capture and strand-specific library preparation. The library quantities were measured by Quant-iT 1× dsDNA HS Assay kit (Thermo Fisher Scientific) with Fluostar Omega (BMG Labtech, Ortenberg, Germany). The fragment size distribution of the libraries was determined by capillary electrophoresis on Labchip GX Touch Nucleic Acid Analyzer on XMark HT chip by using DNA NGS 3k Assay kit (Perkin Elmer). Pooled libraries were sequenced with 20M 150 bp paired-end reads on the NovaSeq 6000 platform (Illumina, San Diego, CA, USA).

### 4.6. Bioinformatic Analysis Process

The fastq files from Illumina were first trimmed for adapter sequence and low-base call quality (Phred score < 30 at ends) with Trimmomatic (v. 0.39) [35] and subsequently assessed for quality by using the FastQC tool (v. 0.12.1, http://www.bioinformatics.babraham.ac.uk/projects/fastqc/, accessed on 11 April 2023) [36]. HISAT2 (v. 2.2.1) [37] was used to map the trimmed reads and to produce the BAM files in genomic coordinates. GRCh38 was used as the reference genome. Gene-level quantifications were calculated using featureCounts (v. 2.1.1) [38]. Trimming, alignment, and gene expression quantification were performed using the Galaxy platform (v. 25.0) [39].

Differential gene expression analysis was performed using the DESeq2 package (v. 1.48.0) [40], and differentially expressed protein-coding genes were considered significant if their *p*-value after Bonferroni correction was <0.05. All the differential analyses were performed by using R (v. 4.5.1). The raw data have been deposited on Gene Expression Omnibus (GEO Database), Accession Number GSE234510.

### 4.7. Validation of Gene Expression Data by Quantitative-Real Time-PCR

Quantitative real-time polymerase chain reaction (RT-PCR) analysis was performed using an ABI Prism 7300 (Applied Biosystems, Thermo Fisher Scientific, Waltham, MA, USA). Amplification was carried out using SensimixPlus SYBR Master mix (Bioline). Primers (Table 1), synthesized by Bio-Fab research, were designed using Primer Express software v1.4.0 (Applied Biosystems). Data were analyzed by the 2^−ΔΔCt^ method, determining the transcript abundance relative to the 18S housekeeping gene.

### 4.8. siRNA Transfections

A previously selected probe (Fw 5′-GUGCCUACAUCCAUGAUGUTT-3′, Rv 5′-ACAUCAUGGAUGUAGGCACTT-3′) among four different siRNA oligos targeting *PI-PLC γ1* [9], designed by GenePharma (Zhangjiang Hi-Tech Park, Shanghai, China), was transfected into human primary FLSs in 24-well plates, using DreamFect Gold (OzBiosciences, Marseille, France). Briefly, a final concentration of 50 nM of each siRNA was diluted in Opti-MEM (Gibco, Thermo Fisher Scientific, Waltham, MA, USA, Cat. 31,985,062), followed by the addition of 5 μL/sample of DreamFect Gold (Cat. DG80500). After incubation for 15 min, the mix was added to 7.5 × 10^4^ cells/0.5 mL in each well in DMEM supplemented with 10% FBS and allowed to culture for 24 h at 37 °C in a CO_2_ incubator. To verify the effectiveness of silencing, transfected cells with the siRNA oligo were analyzed by RT-PCR [9].

### 4.9. CELF1 and PTBP3 Transfections

To perform the transfection experiment, 7 × 10^4^ FLSs were seeded in 24-well culture devices. The reaction was performed in Opti-MEM medium (Gibco, Cat. 31,985,062), with 0.5 µg of pCMV3 vector containing human CELF1 transcript variant 3 (Cat # HG11523-UT, Sino Biological, Eschborn, Germany) or 0.5 µg of pCMV3 vector containing human PTBP3 (Cat # HG21894-UT, Sino Biological), using DreamFect Gold (OzBiosciences, Marseille, France). After 15 min, the reaction mix was added to the cells and left for 24 h and 48 h; then, mRNA was extracted and analyzed by RT-PCR.

### 4.10. Statistical Analysis

All data, regarding RT-PCR, were obtained from at least three independent experiments, each performed either in duplicate or in triplicate, as specified. Data were statistically analyzed with two-way repeated measures analysis of variance (ANOVA) with Bonferroni’s multiple comparison test, using Prism 5.0 software (GraphPad Software, San Diego, CA, USA). The difference was considered statistically significant if the *p*-value, corrected in case of multiple tests, was less than 0.05.

## Figures and Tables

**Table 1:** List of primers used for RT-PCR. The accession numbers are indicated.

Gene	Primer Sequences (Fw-Rv)
*CELF1* NM_001376376	5′-ATCCCTGTCTCTTCAGCTTC-3′ 5′-GCATCAAAGGTCAACACAAGG-3′
*ERN1* NM_001433	5′-AGGTGGTTCTGTCAGAGATC-3′ 5′-AAGTAGCACACGAAGTCGTC-3′
*RBM47* NM_001098634	5′-TGGAAATCCCTACCGTCAAC-3′ 5′-GGAAACATGGAATACTGAGC-3′
*WTAP* NM_001270531	5′-AGTGCCTGGAAGTTTACGCC-3′ 5′-TAAGCATTCGACACTTCGCC-3′
*PTBP3* NM_001163788	5′-ATTTATGCCTGCCTGTTCCC-3′ 5′-TGAGCAGAGGCATTCATAGG-3′
*PSIP1* NM_033222	5′-AGATCAAGGGAAGAAAGGGC-3′ 5′-TCTTGAGCATCAGATCCTCC-3′
*PI-PLCG1* NM_182811.2	5′-CTACCTGGAGGACCCTGTGAAC-3′ 5′-ATCCTCGTTGCCCTGGTCACTG-3′
18S NM_003286	5′-CGCCGCTAGAGGTGAAATTC-3′ 5′-CATTCTTGGCAAATGCTTTCG-3′

## Data Availability

Data are contained within the article and are available upon request.

## Supplementary Materials

The following supporting information can be downloaded at: https://www.mdpi.com/article/10.3390/ijms26178123/s1.
