# Supplementary material for: Molecular Basis of Intron Retention in PI-PLC γ1 mRNA from Osteoarthritis Synoviocytes
Source: Int J Mol Sci. 2025 Aug 22;26(17):8123. doi: 10.3390/ijms26178123 (PMC12427997; doi:10.3390/ijms26178123)
Supplement: Supplementary file 1 [file ijms-26-08123-s001.zip › ijms-3796682-supplementary.pdf]

Supplementary results

Table S1. Patients who underwent total hip and knee arthroplasty surgery. The tissues isolated from these patients were analyzed to verify whether PI-PLC γ1 mRNA had correct splicing (WT) or Intron Retention (IR)

Sample	Name	Age	Gender	Joint	PI-PLC γ1
**1**	D.C	68	♂	Hip	WT
**2**	ME.Z.	82	♀	Knee	WT
**3**	Q.C.	73	♂	Hip	WT
**4**	C.B	69	♂	Hip	WT
**5**	AM. G.	74	♀	Knee	IR
**6**	G.T.	74	♂	Knee	IR
**7**	MR. M.	81	♀	Knee	IR
**8**	C.T.	62	♀	Knee	IR
**9**	M.P.	74	♂	Hip	IR
**10**	R.F.	81	♀	Knee	WT
**11**	DU.G.	63	♂	Hip	IR
**12**	A.T.	85	♀	Knee	WT
**13**	A.S.	87	♀	Knee	WT
**14**	F.C.	79	♀	Knee	IR
**15**	C.C.	88	♀	Knee	WT
**16**	E.S.	70	♀	Knee	WT
**17**	I.M.	80	♀	Knee	WT
**18**	A.C.	81	♀	Knee	IR
**19**	P.A.	76	♀	Knee	WT
**20**	M.T.	79	♂	Knee	IR
**21**	R.L.	82	♀	Knee	IR
**22**	A.M.	80	♂	Knee	WT
**23**	L.L	69	♀	Knee	WT
**24**	C.F.	69	♀	Knee	WT
**25**	G.C.	78	♂	Hip	WT
**26**	V.C.	77	♂	Hip	IR
**27**	F.V.	71	♀	Knee	WT
**28**	D.D.	72	♂	Hip	WT
**29**	R.M.	80	♂	Knee	IR
**30**	A.S.	78	♂	Hip	IR
**Summary**					
**Sample**		**Average Age**	**Gender**	**Joint**	**PI-PLC γ1**
30 patients		76.067 ± 6.544	♂= 13 (43.33%)<br>♀=17 (56.67%)	Knee= 21 (70%)<br>Hip =9 (30%)	WT=17 (56.67%)<br>IR=13 (43.33%)
**Summary- IR samples**					
**Sample**		**Average Age**	**Gender**	**Joint**	
13 patients		75.692 ± 6.459	♂= 6 (46.15%)<br>♀=7 (53.85%)	IR in Knee=9 (69.23%)<br>IR in Hip=4 (30.77%)	
**Summary- WT samples**					
**Sample**		**Average Age**	**Gender**	**Joint**	
17 patients		76.352 ± 6.791	♂= 5	IR in Knee=12 (70.59%)	

		(29.41%)<br>♀=12<br>(70.59%)	IR in Hip=5 (29.41%)

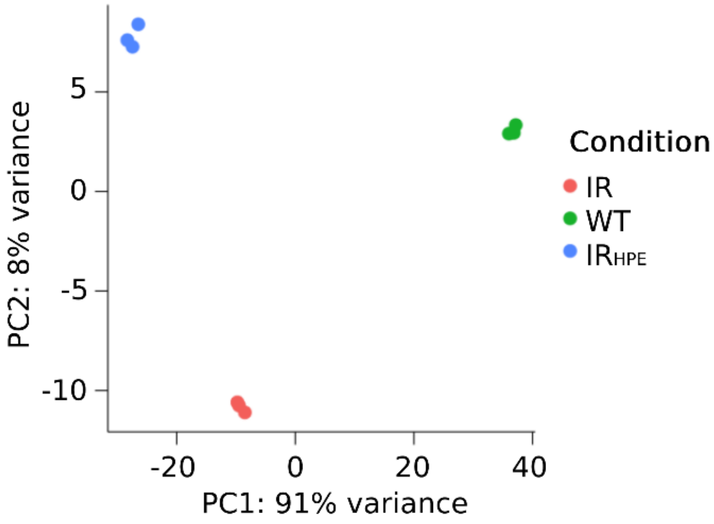

Figure S1. Principal Component Analysis PCA obtained by RNA-seq experiment. PCA score plot of PC1 and PC2 from sample did not show Intron Retention phenomenon (WT, green dot), sample showing IR phenomenon (IR, red dot) and sample showing IR phenomenon and treated with HPE (IRHPE, blue dot). PC1 and PC2 explained 91% and 8%, respectively, of the total variation across the data sets.

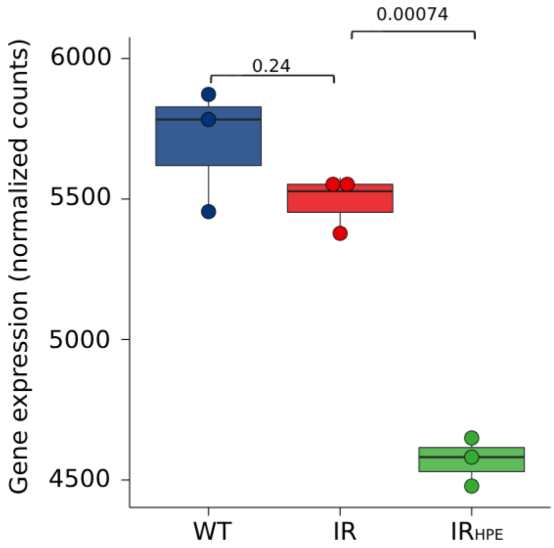

Figure S2. PLCG1 expression in wild type sample (WT), IR sample (IR) and HPE-treated IR sample (IRHPE). RNA-seq analysis was performed on sample did not show Intron Retention phenomenon (WT), sample showing IR phenomenon (IR) and sample showing IR phenomenon and treated with HPE (IRHPE). Data were obtained by a triplicate for each sample; the difference of *PI-PLC γ1* expression was not statistically significant between WT and IR (p=0,24), whereas the difference was statistically significant between WT and IRHPE (p=0.00074).
